# Could erectile dysfunction be a side effect of CGRP inhibition? A case report

**DOI:** 10.1177/03331024211037304

**Published:** 2021-08-18

**Authors:** Linda Al-Hassany, Tessa de Vries, Johannes A Carpay, Antoinette MaassenVanDenBrink

**Affiliations:** 1 Division of Vascular Medicine and Pharmacology, Department of Internal Medicine, Erasmus MC University Medical Center, Rotterdam, The Netherlands.; 2 Department of Neurology, Tergooi Hospital, Hilversum, The Netherlands.

**Keywords:** Case report, erectile dysfunction, galcanezumab, CGRP inhibition, migraine, side effect, adverse event

## Abstract

**Background:**

Recently, antimigraine drugs targeting the calcitonin gene-related peptide pathway have been approved for clinical use as preventive migraine medication.

**Case report:**

We present a case of a 54-year-old male migraine patient, who reported erectile dysfunction as a possible side effect of treatment with galcanezumab, a monoclonal antibody targeting calcitonin gene-related peptide. His potency recovered after treatment discontinuation.

**Discussion:**

As calcitonin gene-related peptide is involved in mammalian penile erection, erectile dysfunction is a conceivable side effect associated with calcitonin gene-related peptide inhibition. Postmarketing surveillance will elucidate the actual incidence of erectile dysfunction in patients using these new antimigraine drugs, and determine whether a causal relationship between calcitonin gene-related peptide inhibition and erectile dysfunction exists. This would be relevant not only because of the direct sexual consequences of erectile dysfunction, but also considering the potential cardiovascular consequences of calcitonin gene-related peptide (receptor) blockade and the association of both migraine and erectile dysfunction with cardiovascular disease.

**Conclusion:**

Erectile dysfunction might be an overlooked, but reversible side effect in male migraine patients using monoclonal antibodies that inhibit the calcitonin gene-related peptide pathway, including galcanezumab. This paper may raise clinical awareness and suggest that this potential side effect needs to be studied further.

## Introduction

Migraine is considered one of the most disabling neurovascular disorders, and many migraine patients receive preventive treatment (1,2). Recently, two classes of migraine-specific drugs have been introduced, which either directly target the neuropeptide calcitonin gene-related peptide (CGRP) or its receptor, namely *gepants* and *monoclonal antibodies* (3). The latter group comprises of erenumab – which targets the canonical CGRP receptor – and fremanezumab, galcanezumab, and eptinezumab – which target the CGRP ligand. All four monoclonal antibodies have been approved by both the United States Food and Drug Administration (FDA) and European Medicines Agency (EMA) (except for eptinezumab, which is currently being reviewed by the EMA) for the prophylaxis of migraine with and without aura in adults (4,5). Yet, clinical trials do not necessarily reflect real-life outcomes of the safety and tolerability of drugs, highlighting the need for real-world insights and experiences on the management of migraine.

Therefore, we present a case of a 54-year-old male patient with migraine, who reported erectile dysfunction as a possible side effect of galcanezumab (Emgality®).

## Clinical case

A 54-year-old male Caucasian patient of normal body weight (BMI 22 kg/m^2^) received subcutaneous injections of galcanezumab for the preventive treatment of migraine attacks. He suffered from migraine with aura since his childhood.

The patient used subcutaneous sumatriptan 6 mg and frovatriptan 2.5 mg tablets as needed for the acute treatment of his migraine attacks. Past preventive migraine medication included beta-blockers, candesartan and topiramate. Given the history of failure of three preventive migraine drugs and a burden of six monthly migraine days, the patient was administered galcanezumab, starting with a subcutaneous loading dosage of 240 mg and 120 mg each month thereafter. Within weeks after starting galcanezumab, the patient reported a complete remission of his migraine. However, he also noted symptoms of erectile dysfunction, that occurred after receiving 120 mg galcanezumab treatment the second time – thus, more than two months after initiating galcanezumab treatment. Because a causal relationship between the erectile dysfunction and the use of galcanezumab was considered possible, we decided to discontinue the galcanezumab injections. Within two months after discontinuation of galcanezumab, corresponding to after approximately two times its half-life, the patient reported that his potency had recovered to a level prior to use of galcanezumab. His general practitioner did not find any plausible (other) explanation for this temporary erectile dysfunction and did not consider it necessary to refer the patient to a specialist (urologist). On rare occasions he used metoprolol for palpitations. In addition, the patient never suffered from erectile dysfunction before, and this was his first episode. Beside erectile dysfunction, he reported no other side effects. No depression, anxiety or other psychosocial comorbidities have been diagnosed or suspected.

## Discussion

This case report raises the question whether erectile dysfunction could be linked to the CGRP inhibiting effect of galcanezumab. In this case, the reversibility of erectile dysfunction after cessation of galcanezumab treatment hints towards a causal association between the use of galcanezumab and erectile dysfunction. Although the patient used metoprolol, if needed, for the treatment of palpitations, we assume it is unlikely that this medication could have triggered or altered his erectile dysfunction. Indeed, he had only taken one tablet on one single day during the entire episode of the erectile dysfunction. Therefore, we believe that no correlation exists with the onset of his erectile dysfunction.

Galcanezumab is a humanized IgG4 monoclonal antibody which targets CGRP and, therefore, prohibits binding of CGRP to its receptor. It has demonstrated its efficacy in both episodic and chronic migraine patients (6). Moreover, a recently published phase 3b multicenter trial, CONQUER, demonstrated that galcanezumab was superior and safe compared to placebo in migraine patients in whom previous standard-of-care preventive medication was not effective (7). Besides, it has been shown to be an effective prophylaxis in patients suffering from episodic cluster headache (8).

Few serious adverse events (SAEs) were reported in these trials (below 2%), and most of adverse events (AEs) were mild to moderate. The most common AEs which were significantly more often reported in galcanezumab versus placebo users include: injection site reaction, including erythema and pruritus, and upper respiratory tract infection (9). However, clinical trials are powered to detect differences in efficacy rather than AEs between the two arms. Besides, men are typically underrepresented in migraine trials considering the higher prevalence of migraine in women (10). Therefore, it is of importance to monitor AEs of new drugs in real-life settings, when possible with a more active approach toward known or potential adverse effects. For example, reduction of erectile function may not be reported spontaneously.

From a theoretical perspective, male erectile dysfunction or impotence is a conceivable side effect associated with blockade of the actions of CGRP. CGRP is an ubiquitous and potent vasodilator, which – besides its role in the migraine pathophysiology – is involved in mammalian penile erection (11).

**Figure 1.**
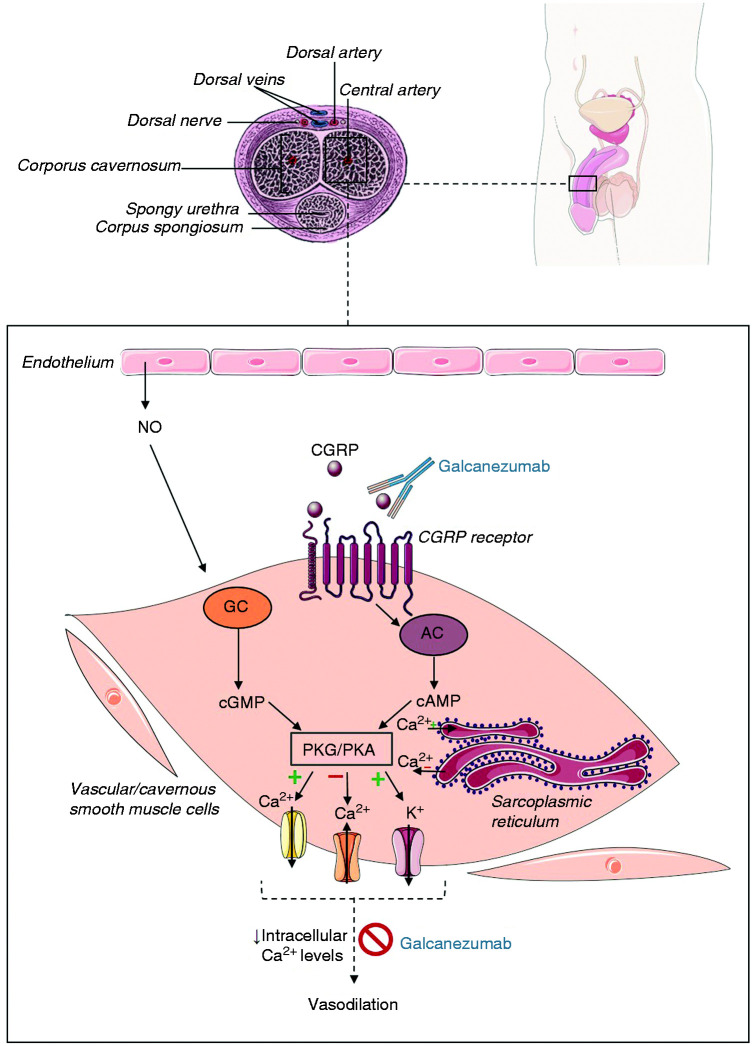
A cross-section of the human penis is depicted, indicating its anatomical structures, as well as the target and effects of a monoclonal antibody targeting CGRP, e.g. galcanezumab. The penile erection involves a decrease in venous outflow and dilation of arteries within the corpora cavernosa due to relaxation of corporal smooth muscle. The latter mechanism involves a dual action of both NO, released from nonadrenergic-noncholinergic neurotransmission and the endothelium, and CGRP. This leads to protein kinase G, or cGMP-dependent protein kinase (PKG) and protein kinase A, or cAMP-dependent protein kinase (PKA) activation due to increased concentrations of cGMP and cAMP, respectively, within the smooth muscle cells. Subsequently, hyperpolarization due to K^+^ efflux and a decrease in intracellular Ca^2+^ levels lead to vasodilation. Galcanezumab targets CGRP and might impede this vasodilatory response, which occurs in response to sexual stimuli, leading to erectile dysfunction (25–27).

Penile erection involves relaxation of the smooth muscle cells of the corpora cavernosa, which is primarily mediated by nitric oxide (NO) via accumulation of cyclic guanosine monophosphate (cGMP) – a process controlled by neuroregulation (12). CGRP also contributes to regulation of the penile erection and is present within sensory nerves near the cavernous artery, and within the arterial walls and the smooth muscle cells of the cavernous artery. Besides, it has been demonstrated that the autonomic nervous system (sympathetic) fibers may express CGRP, in coexpression with other factors, such as vasoactive intestinal polypeptide (VIP) and NO synthase (13). Intracavernous injection of CGRP in impotent males has been shown to induce relaxation of the smooth muscle cells of the corpora cavernosa, and an increase in the arterial inflow and veno-occlusion of the cavernous outflow, leading to an erection (11). In an earlier pilot study, responses to intracavernous injection of a CGRP antibody were investigated in monkeys. Administration of this CGRP antibody (500 ng) did not alter erectile responses to neurostimulation, probably due to the short time course of the experiment, not permitting sufficient time for the antibody to exert its function by reaching its relevant site of action, as recently reviewed by Hargreaves and Olesen (14). Yet, intracavernous administration of CGRP led to an erectile response by increasing the arterial flow and inducing occlusion of the venous outflow as well as relaxation of the cavernous smooth muscle. The latter responses still suggest a role for CGRP as one of the key neurotransmitters in erections (15). CGRP has been reported to exert its effect via hyperpolarization through the efflux of potassium, due to an increase of intracellular cyclic adenosine monophosphate (cAMP), which ultimately leads to a decrease in intracellular Ca^2+^ levels (Figure 1) (16). Considering its physiological effects in penile erection, Deng et al. studied CGRP as a target for erectile dysfunction treatment. They applied adenoviral-mediated gene transfer of CGRP into the corpora cavernosa in aged rats with erectile dysfunction, which showed physiological improvement (17).

Epidemiological studies have shown an increased risk of cardiovascular events in men with erectile dysfunction, which might serve as an early marker and predictor for (cardio)vascular disease, as reviewed by Mobley et al. (18). This association is probably driven by endothelial dysfunction and reduced production of NO.

In this respect, caution might be advised for certain users of these monoclonal antibodies that inhibit the CGRP pathway, considering (i) the compensatory role of CGRP in cardiovascular conditions, including ischemia, and the potential long-term cardiovascular disadvantages due to its blockade (19), (ii) the fact that besides erectile dysfunction, migraine – particularly with aura – is associated with significant cardiovascular sequelae, including myocardial infarction and ischemic stroke, in both sexes (20,21), and (iii) earlier findings which indicate a correlation between migraine and erectile dysfunction (22,23). Yet, we underline that we aim to raise awareness with this single case, rather than to prevent caregivers from prescribing monoclonal antibodies as antimigraine agents, especially to young healthy men.

Therefore, we suggest that clinicians pay attention to the occurrence of erectile dysfunction as a potential side effect of (prophylactic) antimigraine drugs which target CGRP or its receptor, namely gepants and monoclonal antibodies (including galcanezumab). In patients with this potential side effect, especially when no other plausible explanation for their impotence is found, we recommend that a causal relationship is explored further by stopping the administration of the antimigraine drug. In addition, further studies are warranted to relate this potential side effect to CGRP levels in plasma, assessed longitudinally before, within and after the therapy – although systemic levels are not necessarily completely indicative for locally released CGRP. Finally, a screening and cardiovascular follow-up of migraine patients with established erectile dysfunction after these antimigraine drugs might be recommended in view of the relationship between erectile dysfunction and cardiovascular disease. While the burden of migraine should definitely not be overlooked, attention should be also paid to the influence of erectile dysfunction as a possible side effect on the quality of life in male patients (24). Yet, in this respect, we want to emphasize that the first step would be to conduct further research to either confirm or refute a causal relationship between these antimigraine drugs and erectile dysfunction, which cannot be established based on this single report.

## Conclusion

Erectile dysfunction in male migraine sufferers who use monoclonal antibodies that inhibit CGRP activity, including galcanezumab, might be an overlooked and underreported or rare, but reversible, side effect. We bring this case to prompt clinical awareness, although we realize that more data on the use of these preventive therapies in migraine are warranted to further substantiate the incidence and potential (cardiovascular) severity of this potential side effect in migraine patients.

## Clinical implications

- Now that monoclonal antibodies that inhibit CGRP activity are approved for the preventive treatment of migraine, their safety should be monitored in real-world clinical settings.
- The presented case report of a male migraine patient raises the hypothesis that erectile dysfunction might be a side effect of monoclonal antibodies which antagonize CGRP.
